# Optimal site selection and image fusion guidance technology to facilitate cardiac resynchronization therapy

**DOI:** 10.1080/17434440.2018.1502084

**Published:** 2018-07-30

**Authors:** Benjamin J. Sieniewicz, Justin Gould, Bradley Porter, Baldeep S Sidhu, Jonathan M Behar, Simon Claridge, Steve Niederer, Christopher A. Rinaldi

**Affiliations:** a Division of Imaging Sciences and Biomedical Engineering, King’s College London, London, United Kingdom; b Cardiology Department, Guys and St Thomas’ NHS Foundation Trust, London, United Kingdom

**Keywords:** CRT, site selection, heart failure, image fusion, image guidance, electrical latency, mechanical latency, tissue viability

## Abstract

**Introduction**: Cardiac resynchronization therapy (CRT) has emerged as one of the few effective treatments for heart failure. However, up to 50% of patients derive no benefit. Suboptimal left ventricle (LV) lead position is a potential cause of poor outcomes while targeted lead deployment has been associated with enhanced response rates. Image-fusion guidance systems represent a novel approach to CRT delivery, allowing physicians to both accurately track and target a specific location during LV lead deployment.

**Areas covered**: This review will provide a comprehensive evaluation of how to define the optimal pacing site. We will evaluate the evidence for delivering targeted LV stimulation at sites displaying favorable viability or advantageous mechanical or electrical properties. Finally, we will evaluate several emerging image-fusion guidance systems which aim to facilitate optimal site selection during CRT.

**Expert commentary**: Targeted LV lead deployment is associated with reductions in morbidity and mortality. Assessment of tissue characterization and electrical latency are critical and can be achieved in a number of ways. Ultimately, the constraints of coronary sinus anatomy have forced the exploration of novel means of delivering CRT including endocardial pacing which hold promise for the future of CRT delivery.

## 1. Introduction

One in five people will suffer from heart failure (HF) during their lifetime and once diagnosed, ~40% of patients die within 1 year [1]. Cardiac resynchronization therapy (CRT), by pacing the left (LV) and right (RV) ventricles to re-coordinate cardiac electrical activation and produce a synchronous contraction, has emerged as one of the few effective treatments for HF [2,3]. However, at present 30% of patients fail to respond clinically through improved quality of life, exercise capacity and New York Heart Association (NYHA) functional classification of HF and up to 50% show no beneficial changes in cardiac function [3]. Suboptimal LV lead position is a common culprit when evaluating poor outcomes after CRT [4–7]. Equally, several groups have reported enhanced response rates when targeting tissue which displays evidence of favorable viability [8–11] or advantageous mechanical [12–18] or electrical [19–23] properties. This review will evaluate how to define the optimal LV pacing site and the mechanisms by which it is possible to selectively deploy a pacing electrode at this site.

## 2. Acute and chronic markers of response

In order to identify the optimal pacing location, it is necessary to perform an examination of the available sites and preferentially select the site which possesses the most favorable characteristics. Unfortunately, in the 20 years since the first description of resynchronization pacing for HF [24], no consensus has been achieved on how to define ‘response’ to CRT [25] making comparison of the various pacing sites problematic. A multitude of different clinical and event-based definitions of response to CRT have been described with rates of response varying from 32% to 91% depending on the criteria used. This review will predominantly focus on three indices; outcome-based metrics which evaluate survival and mortality after device implantation. Markers of left ventricular reverse remodeling (LVRR) following device implantation, of which a reduction in LV end-systolic volume (LVESV) is the most widely accepted marker [26,27]. Finally, some metrics evaluate acute changes in LV contractility. Acute hemodynamic response (AHR) is a reproducible marker of LV contractility best expressed as the change in the maximum rate of left ventricular pressure (LV-dP/dt_max_), from a baseline control state [28,29]. Previous work has evaluated the acute hemodynamic effects of CRT using LV-dP/dt_max_ as an outcome measure [29–32], and this metric has been used to compare the effects of biventricular (BiV) pacing at different locations [28,29,33]. An improvement in LV-dP/dt_max_ of 10% during acute implantation has been shown to predict chronic LV reverse remodeling in patients receiving CRT [34].

## 3. Tissue characterization

### 3.1. Pathophysiology of scar in ischemic and non-ischemic cardiomyopathy

Ischaemic scar forms as a result of permanent myocyte death following an ischaemic insult. A reparative process is initiated to rebuild the infarcted myocardium and maintain the structural integrity of the ventricle. An initial inflammatory phase of healing is followed by a fibrogenic phase that eventually results in the formation of scar tissue as dead myocytes are progressively replaced by collagenous scar. Ischaemic scar tends to display a sub-endocardial or transmural distribution affecting a specific coronary territory. Histological evidence of myocardial fibrosis has also been described in non-ischaemic presentations [35–37]. While the precise mechanism behind this scar formation, which typically follows an epicardial or mid-will distribution, is unclear, interstitial and perivascular fibrosis ultimately results in myocardial necrosis [38].

### 3.2. Impact of scar on the mechanical properties of the heart

Almost immediately following coronary artery occlusion, the subtended area of myocardium becomes passive and non-contractile. The non-viability and reduced plasticity of infarcted scar tissue is associated with a reduction in efficient and effective mechanical function during systole. Nearly all of the determinants of systolic function are negatively impacted by the presence of scar including cardiac shape and dimensions, preload, afterload and contractility [39]. During systole, the scared region stretches and bulges outward while the remaining myocardium contracts, causing a reduction in the mechanical efficiency of the heart as a pump. This effect is strongly dependent on the total area of scared tissue [40].

### 3.3. Impact of scar on outcomes

The size, location, and transmurality of scar all impact LV remodeling after CRT. Global scar burden has been shown to be inversely proportional to LV reverse remodeling amongst both ischemics [41], non-ischemics [42], and mixed populations [43–45]. Functional improvement [41,42,46] and survival [47] are also inversely proportional to scar burden. Retrospective analysis has confirmed that favorable markers for response include smaller scar size and fewer areas of transmural scar [45]. The location of scar is of equal importance, in particular when it is located in the posterolateral region of the LV, a site empirically thought to be optimal for LV lead deployment. Scar in this area is associated with lower response rates following CRT [45,48].

### 3.4. Impact of scar on electrical activation

Scar prevents effective transmission of the electrical impulse, resulting in prolonged activation. Electrical activation in regions of fibrosis is characterized by localized delays and fractionated, low-amplitude extracellular electrograms [49]. This has been attributed to changes in patterns of excitation and conduction due to altered ion channel activity [50] and decreased cellular connectivity [51] compounded by tortuous conduction through areas of surviving myocytes. This delay in LV activation results in less hemodynamic improvement during BiV pacing [52]. Electrical stimulation in regions of scar can also be pro-arrhythmic [53,54] and is associated with increased morbidity and mortality [8,55]. Unsurprisingly, the presence of myocardial scar at the site of LV stimulation during CRT is associated with non-response [8,56].

### 3.5. Scar identification

Given the negative implications associated with stimulating scared and fibrotic myocardial tissue, current evidence favors avoiding these areas and targeting viable tissue. Numerous mechanisms have been proposed in order to differentiate non-viable tissue, and these can be categorized into anatomical, functional, and biological modalities, see Table 1. Anatomical imaging involves direct visualization of tissue defined as scarred. Functional imaging relies on surrogate markers of scar such as measures of wall motion, strain, voltage, or contractile reserve. Biological imaging assesses metabolism or perfusion as a surrogate for viability.

**Table 1.** Techniques for assessment of viability and scar.

Imaging technique	Classification	Technique	Marker of viability/scar
CMR	Anatomical imaging	LGE	Direct visualization of scar
	Anatomical imaging	Wall thickness	EDWT as surrogate for scar
	Functional imaging	Contractile reserve	Contractile reserve
	Functional imaging	Functional assessment	Severe dysfunction as surrogate for scar
	Functional imaging	Strain assessment	Severely reduced strain as surrogate marker
TTE	Anatomical imaging	Wall thickness	EDWT as surrogate for scar
	Functional imaging	Contractile Reserve	Contractile reserve
	Functional imaging	Functional assessment	Severe dysfunction as surrogate for scar
	Functional imaging	Strain assessment	Severely reduced strain as surrogate marker
CT	Anatomical imaging	Wall thickess	EDWT as surrogate for scar
PET or SPECT	Biological imaging	Perfusion	Perfusion as a surrogate for viability
PET or SPECT with FDG	Biological imaging	Glucose utilization	Glucose utilization as a surrogate for viability

CMR: cardiac MRI; LGE: late gadolinium enhancement; CT: computed tomography; PET: positron emission tomography; SPECT: single-photon emission computed tomography; FDG: Fluorine-18-labeled deoxyglucose.

### 3.6. Cardiac MRI

Late gadolinium enhancement (LGE) cardiac MRI (CMR) is the gold standard for delineating myocardial scar with high resolution, as the superior spatial resolution of LGE-CMR permits differentiation between epicardial, transmural, and sub-endocardial infarction. The technique relies on the fact that gadolinium washes out of the blood pool but accumulates in the extracellular space. Tissues with weak intracellular bonds and high amounts of non-cellular space, including necrotic tissue or fibrous scar, will develop higher concentrations of gadolinium than the surrounding healthy tissues. Scar detected by LGE-CMR has been shown to closely match histologically proven myocardial infarction [57].

### 3.7. Trans-thoracic echocardiography

Transthoracic echocardiography has the potential to identify areas of scarred or fibrotic myocardium. Early work focused on regional wall thinning [58] and assessment of regional contractile function [59], while more recent work has focused on the ability of speckle-tracking radial strain [60] and longitudinal strain [61] to better identify areas of regional akinesis. Other techniques include the use of 3D contrast echo [62] and pulse cancelation echocardiography [63], which have both shown some promise as tools to identify areas of scar.

### 3.8. Cardiac computed tomography (CT)

Tissue characterization using cardiac CT has been used to identify areas of myocardial scarring. After an infarct, myocardial tissue replaced by fibrous scar and eventually, after several months, undergoes significant lipomatous metaplasia [64]. Using unenhanced CT, it is possible to identify the fat in infarcted myocardium. New-generation dual-source CT (DSCT) allows the integration of late-iodine enhancement imaging and has been shown to correlate reasonably well (52% sensitivity, 88% specificity) with LGE-derived CMR imaging [65].

### 3.9. Nuclear imaging

Tracer uptake during Nuclear imaging using either positron emission tomography (PET) or single-photon emission CT (SPECT) relies on adequate myocardial blood flow and myocyte viability. The finding of a fixed perfusion defect can either represent myocardial scar or viable hibernating myocardium. Differentiation between these two states can be further enhanced through an assessment of glucose uptake via Fluorine-18-labeled deoxyglucose (FDG) with hypocontractile regions exhibiting reduced perfusion but normal or increased FDG uptake representing likely hibernating myocardium. During head-to-head comparison, MIBI has been shown to consistently overestimate areas of myocardial scar tissue, while FDG lacks the spatial resolution associated with LGE-CMR [66].

### 3.10. Electroanatomical mapping

While LGE-CMR has the capacity to directly visualize anatomic myocardial scar, the abnormal electrophysiological substrate extends beyond the dense anatomical scar, into regions of heterogeneous ‘boarder-zone’ tissue [67], and may be optimally identified using electroanatomic mapping (EAM). The ability of EAM to assess myocardial viability on the basis of myocardial voltage has been validated against SPECT [68], PET imaging [69], and latterly LGE-CMR [70–73] in both ischemic and non-ischemic cardiomyopathies. Furthermore, analysis of electrogram characteristics can also help to predict histologic properties of scar tissue [49].

### 3.11. Invasive electroanatomical mapping

During invasive EAM, intracardiac electrical activation is recorded in relation to anatomic locations in a particular cardiac chamber of interest, allowing the definition of 3D cardiac chamber geometry as well as delineating areas of anatomic interest such as regions of scar. Systems can be divided into contact and non-contact mapping systems. Contact mapping systems rely on recording local activation between two poles on a mapping catheter. The resulting bipolar voltage map can be thresholded to reveal areas with a voltage outside of normal range for ventricular tissue, typically 0.5 mV to 1.5 mV.

Non-contact mapping systems utilize a multi-electrode array (MEA) catheter to simultaneously record endocardial activation over multiple areas [74]. The array is situated on a balloon with 64 electrodes allowing high-density mapping from a single heartbeat. Advantages of this system include the ability to acquire multiple endocardial electrograms during a single cardiac cycle; however, this comes at the cost of greater inaccuracy in electrogram timing and morphology at greater distances from the MEA [75]. Work evaluating this system has already established that non-contact mapping can identify regions of electrically viable myocardium, which could be used to inform lead position, particularly among ischemic patients [52].

### 3.12. Electrocardiographic imaging

**Figure 1.**
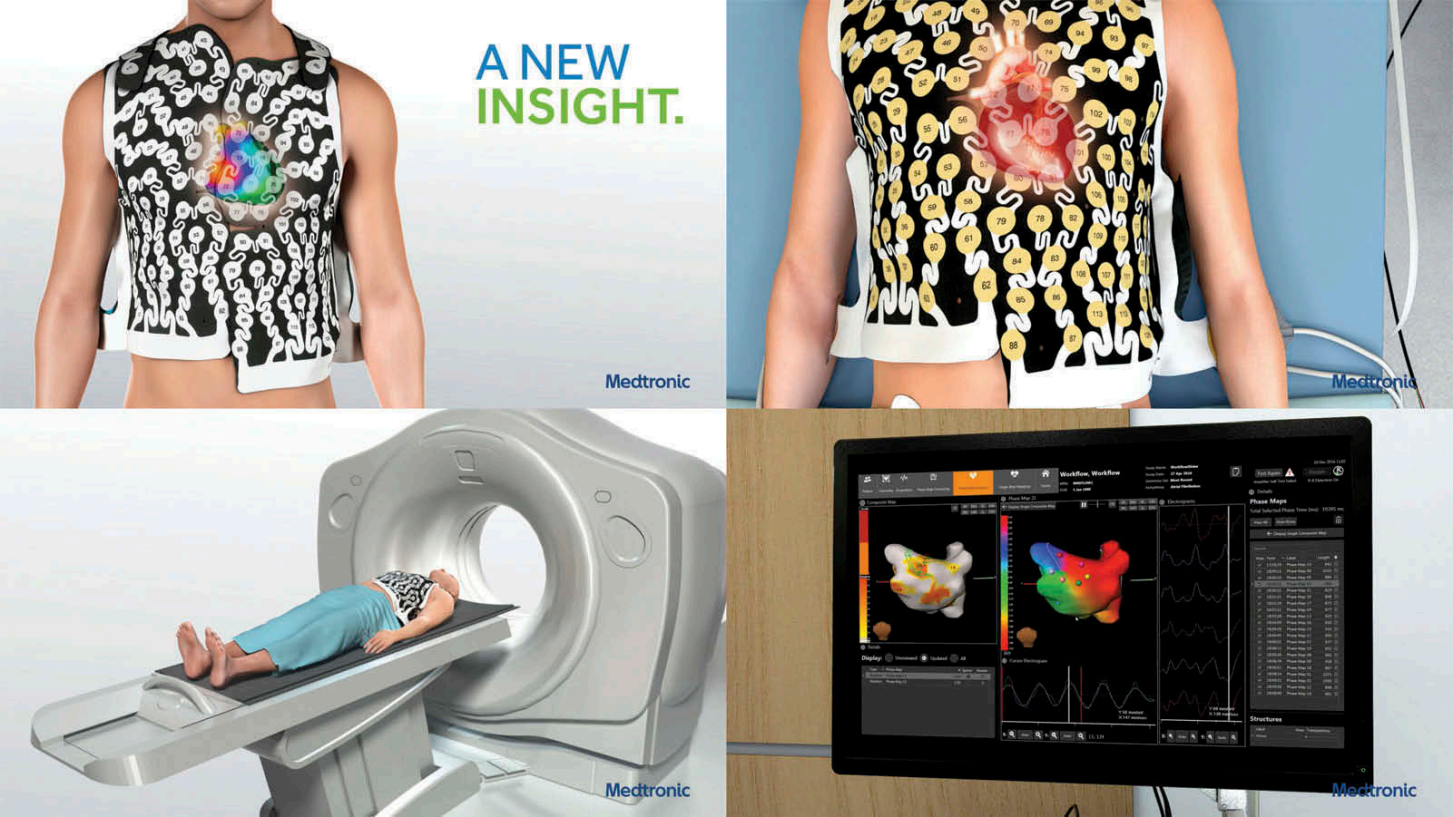
The 252-lead vest records torso surface electrograms. Reproduced with permission from MEDTRONIC.

Electrocardiographic imaging (ECGI) is a novel, non-invasive 3D epicardial electrophysiology imaging modality. This technique uses 252 ECG electrodes mounted on a wearable vest to reconstruct epicardial potentials from torso potentials, see Figure 1. These are displayed as electrograms and activation sequences (isochrones) on the epicardial surface of the heart [76].

ECGI benefits from a non-invasive approach and is able to measure the activation across the whole heart simultaneously compared to the slower sequential mapping with EAM. Inverse ECG mapping technology is able to identify fibrotic tissue due to the abnormal electrical properties exhibited by scarred myocardium, specifically low-amplitude electrical potentials with broad fractionated electrograms typically in areas exhibiting delayed or slow activation [77–79]. A degree of discrepancy between CMR and EAM is expected as CMR can struggle to detect areas of homogenous microscopic diffuse fibrosis due to the low resolution of the image, while inverse ECG EAM can be more sensitive at detecting zones of epicardial and transmural fibrosis but may miss sub-endocardial scar. Despite this, good correlation has been observed between areas of low voltage on ECGI and areas of scar, as identified on LGE-CMR [80] with one study quoting an 89% sensitivity and 85% specificity at detecting epicardial scar [81].

## 4. Dyssynchrony assessment and identification of the site of latest mechanical activation (LMA)

A paradox exists between the electrical substrate corrected by CRT, specifically dyssynchronous BiV electrical activation and its mode of action, which is predominately mechanical and aims to enhance cardiac contractility by correcting the mechanical dyssynchrony and restoring the mechano-energetic efficiency of the heart. Intuitively, it would seem sensible to specifically assess the degree of mechanical dyssynchrony present, as this would both help patient selection and aid in the determination of the optimal LV pacing site. Dyssynchrony is the measure of dispersion in the timing of mechanical contraction of the various LV segments [82] and may be measured by a variety of different imaging techniques.

### 4.1. Transthoracic echocardiography

Early work assessing the utility of echocardiographic parameters of dyssynchrony to aid patient selection for CRT appeared promising. A systolic dyssynchrony index (SDI), calculated from tissue Doppler imaging (TDI), proved capable of retrospectively predicting enhanced clinical response in single-center work [83]. These benefits were also observed in a multicenter retrospective analysis where the use of baseline TDI imaging predicted not only functional and echocardiographic improvement but also identified patients who yielded prognostic benefit from CRT [84]. Speckle-tracking radial strain analysis superseded TDI, as it was less dependent on the angle of incidence of the ultrasound beam and also appeared able to predict echocardiographic response in retrospective analysis [85].

Unfortunately, the utility of mechanical dyssynchrony assessment to identify CRT responders has not been reproduced in larger, prospective, randomized multicenter studies [86] and this has cast some doubt on the reproducibility of the technique. In addition, when all the various echocardiographic measures of mechanical dyssynchrony were analyzed in a large, international multicenter study, no single measure proved capable of improving patient selection for CRT [87]. Promisingly, newer techniques including longitudinal myocardial strain assessment [88] and 3D speckle-tracking echo [89] appear more reliable and indicate an encouraging direction for future work.

Given the primary target of CRT is the restoration of coordinated myocardial contraction and those patients exhibiting mechanical dyssynchrony appeared to yield the most benefit from CRT, it seemed logical that the optimal site for LV lead deployment would be at the site of maximal mechanical delay. In a retrospective analysis where TDI assessment of mechanical activation was performed prior to CRT implantation, patients in whom the LV lead was situated at the site exhibiting the latest activation showed increased functional and echocardiographic improvements [15]. Superior response to CRT was also observed when the site of LMA was targeted using tissue synchronization imaging (TSI) [90], 3D echocardiography [91], and speckle tracking [92]. The TARGET [13] and STARTER [16] trials prospectively assessed the utility of echocardiographic speckle-tracking two-dimensional radial strain imaging to inform LV lead deployment. Echo-guided lead implantation was associated with echocardiographic response rates (>15% reduction in ESV) of 70% and 57%, respectively. Both studies showed increased rates of event-free survival over empirical lead placement.

### 4.2. Cardiac MRI

CMR has several potential advantages when looking to characterize mechanical activation. These include greater reproducibility, less artifact secondary to patient habitus, detailed assessment of myocardial tissue characterization as well as chamber size, and volumes and greater spatial resolution. CMR can also assess strain in multiple planes allowing the assessment of both radial and longitudinal strain. A recent prospective, single-center randomized study (CMR-CRT) showed the feasibility of performing an assessment of circumferential strain in order to identify the latest mechanically activated viable segment [93]. Several dyssynchrony assessment metrics have been proposed including myocardial tagging, displacement encoding with stimulated echoes, and phase contrast tissue velocity mapping. While myocardial tag data has been shown to predict functional improvement following CRT implantation [94], tag decay remains an issue and this led to the development of 3D volumetric change as a means of assessing both global LV dyssynchrony and assessing mechanical activation [95]. When compared to other mechanical dyssynchrony measures, volume changeSDI proved the sole predictor of chronic reverse remodeling [18].

### 4.3. Cardiac CT

Cardiac CT offers a potential benefit over CMR due to the fact that approximately 28% of patients undergoing CRT implantation have already received an implantable cardiac device rendering them unsuitable to undergo CMR scanning [96]. Cardiac CT is associated with submillimeter spatial resolution and can assess regional and global LV dyssynchrony by calculating the stretch of the endocardial surface throughout the cardiac cycle (stretch quantifier for endocardial engraved zones [SQUEEZ]) [97]. When assessed in patients undergoing an upgrade to a CRT pacing system, CT-SQUEEZ targets were associated with a similar improvement in AHR as the best achievable (20.4% ± 13.7% vs. 24.9% ± 11.1%; *P* = 0.36) [98]. In addition, delivering LV stimulation at a site identified using CT-SQUEEZ resulted in greater clinical response vs. non-target segments (90% vs. 60%, *P* < 0.001).

## 5. Identifying the site of latest electrical activation (LEA)

The primary substrate targeted during CRT is delayed electrical activation, typically manifest by a left bundle branch block (LBBB) pattern on the surface ECG. Detailed analysis of ventricular activation confirms a myriad of differing underlying conduction disturbances amongst even this group, with ischemic patients displaying a particularly high degree of variability in activation [99]. The standard 12 lead ECG is therefore of limited use when looking to define the optimal site for LV stimulation, and focus has shifted to more detailed methods of visualizing electrical latency. In the context of LBBB, ventricular activation is initiated at the distal branching of the right bundle, with activation of the left endocardium occurring after a significant delay, as a result of slow conduction through the interventricular septum. Theoretically, the site of latest activation should exhibit the most dyssynchrony and as such would represent an ideal pacing site. While some work appears to confirm the site of LEA is synonymous with the optimal pacing site [21], more recent analysis has shown optimal site exhibits late but not supremely delayed activation [100]. Sites demonstrating excessively delayed activity may in fact merely represent distal activation occurring within islands of non-viable tissue. A variety of different methods of identifying the site of latest activation have been described, as outlined below.

### 5.1. Q-LV and LV lead electrical delay

An advantage of assessing electrical delay is that it can be performed both intra-procedurally and without the need for any additional mapping equipment. Singh et al., devised a measure of electrical latency called the left ventricular lead electrical delay (LVLED) [20]. This marker of electrical delay was calculated during LV lead implantation by determining the onset of the surface ECG-QRS complex to the onset of the sensed electrogram on the LV lead and expressing the value (the Q-LV time) as a percentage of the baseline QRS interval. They identified that LVLED correlated with greater hemodynamic improvements (derived using transthoracic echo).

When dichotomized, patients with an LVLED of >50% exhibited greater event-free survival and reduced rates of hospitalization. In a sub-study of the SMART AV trial [101], patients were again dichotomized, although this time according to the median Q-LV value (95 ms). Gold et al. showed that implanting the LV lead at a site with a favorable Q-LV was independently associated with symptomatic improvement and greater reverse remodeling at 6 months [19].

Both of these studies retrospectively analyzed the degree of electrical latency at the site of LV lead deployment; however, Zanon et al. evaluated whether Q-LV might be used to identify the optimal site in an individual patient by systematically screening all of the suitable coronary sinus (CS) veins [21]. A strong correlation was observed between Q-LV prolongation and improvements in acute hemodynamic response. Again a Q-LV value of greater than 95 ms appeared significant, yielding an improvement in AHR of >10%, a finding which has been associated with predicting long-term remodeling [34]. Crucially, in 96.8% of patients, the optimal hemodynamic performance was associated with delivering pacing therapy at the site exhibiting the LEA. A similar figure (85%) was observed by van Gelder et al. in their evaluation of the effects of LV endocardial pacing amongst a cohort of non-responders to epicardial CRT [102]. The small discrepancy may be attributed to the larger cohort of ischemic patients in this study.

### 5.2. Narrowing of the paced QRS

Reductions in the paced QRS duration (QRSd) during BiV pacing may also aid identification of late activating tissue. Widening of the QRS after CRT implantation has been found to be an independent predictor of mortality or progression to heart transplantation[103], and achieving a reduction in the paced QRS has been shown to predict response in several studies [104] including via multivariate logistic regression [105]. In other work, a reduction in paced QRSd was found to be the only predictor of response [106]. However, this finding is disputed in other studies [107]. There is also no consensus as to whether delivering BiV pacing at a site which achieves a narrowing of the paced QRS is associated with improvements in hemodynamics. While some work has shown a correlation between narrowing of the QRS and improvements in AHR [100], this finding has not been consistently replicated [21].

### 5.3. Invasive electroanatomical mapping

Electroanatomical mapping has also been used to evaluate electrical activation and locate the site of LEA. Analysis of contact and non-contact mapping first data identified a ‘U-shaped’ pattern of activation during LBBB with depolarization originating at a single septal breakthrough site [99]. Activation could not proceed directly from the anterior to the lateral wall, due to the presence of lines of block, forcing the depolarization wave front to pass inferiorly around the apex. Crucially, even amongst patients who presented with LBBB on their surface ECG, the location of this line of block varied between patients, exposing the heterogeneity of this complex conduction disorder and the difficulty in establishing a universal site of LEA.

More detailed analysis of LV activation revealed heterogeneity in conduction velocities in both non-ischemic and ischemic patients at the site of LV stimulation in the lateral and posterolateral walls [52]. The location of these areas of slow conduction influenced the pattern and direction of wave front propagation. While it was possible to mitigate the effects of positioning the lead in an area of slow conduction by altering the timing between LV and RV stimulation during CRT, locating and stimulating healthy, late-activating tissue was consistently associated with superior hemodynamic improvements.

The anatomical constraints of transvenous, epicardial CRT mean that LV stimulation can only occur at a site accessible via a tributary of the CS. Coronary venous electroanatomical mapping allows the assessment of electrical latency exclusively within the CS [23]. A high degree of variability in the location of the site of LEA was observed between patients. Intra-procedural assessment of latency utilizing this technique is feasible and, while of practical value to the implanting physician, is limited to only those sites accessible via the available coronary venous system.

### 5.4. Non-invasive electroanatomical mapping of electrical activation

The heterogeneous nature of LV activation in patients with LBBB has also been described using ECGI. A key advantage of this technique is the ability to non-invasively identify the area of LEA, and this approach has already been shown to allow peri-procedural guidance of the LV lead to the target site. ECGI can also compute an LV electrical dyssynchrony index, and this metric appears may predict patients likely to respond to CRT and aid in identifying the optimal site during LV lead deployment [108,109].

### 5.5. Correlation between the site of LMA and LEA

**Figure 2.**
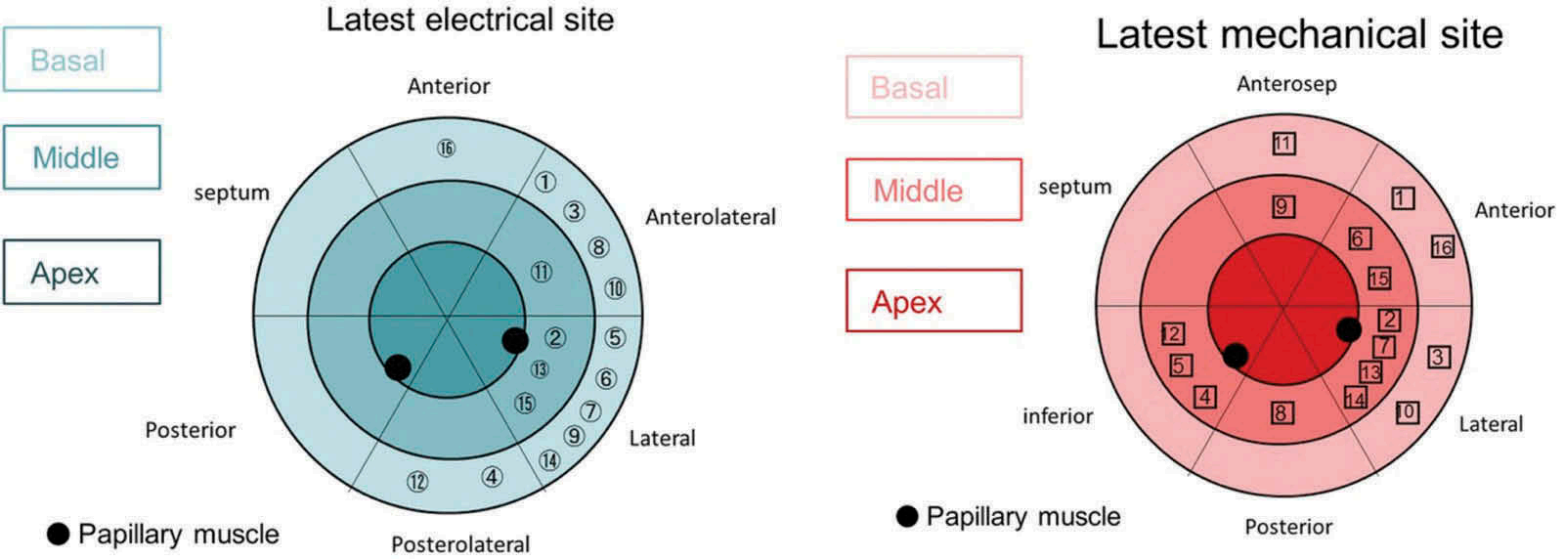
Comparison of sites of LEA (A) and LMA (B). The circled numbers refer to the patient numbers. Reproduced with permission from Wiley.

One hypothesis advanced to explain the persistent issue of non-response to CRT is the existence of uncoupling of mechanical and electrical synchronicity. While these two substrates can be assessed individually, performing an assessment of both may be preferable. Early work appeared to suggest that the site of LEA was synonymous with the area of LMA, when evaluated using non-contact EAM and TTE TDI [110]. Similar findings were observed when the LMA was assessed using CMR [111]. One explanation for this uniformity may be the crucial role played by etiology and the disruptive effects of tissue heterogeneity. The impact of etiology was better assessed by Fujiwara et al. who included patients with both ischemic and non-ischemic cardiomyopathy in their study and identified a clear discrepancy between the site of LMA and LEA [112], see Figure 2. Unanimity between the LMA and the LEA site was only observed in 19% of patients.

### 5.6. Electrical activation during RV pacing and LBBB

Current class 1 indications for CRT include evidence of dyssynchronous electrical activation, manifest on the surface ECG by LBBB. While there has been a great deal of focus on characterizing the precise nature of LV depolarization which occurs during LBBB, activation during CRT occurs as a result of paced stimulation at the LV and typically, the RV apex. While an RV paced event appears morphologically similar on the surface ECG to conduction resulting from LBBB, Eschalier et al. set out to perform a more detailed examination. Non-invasive body surface mapping demonstrated clear differences in the depolarization pattern of both the RV and LV during RV pacing and LBBB activation. Apical pacing resulted in slower RV conduction with lines of conduction detected around the pacing site [113]. LV depolarization was also observed to be prolonged by RV pacing although lines of slow conduction were fewer and shorter in size in comparison to those observed during LBBB activation. The LV site of LEA was similar during both RV apical pacing and LBBB activation and was consistently located at the LV base.

### 5.7. Electrical activation and RV pacing site selection

RV pacing appears to result in delayed LV depolarization and the deleterious effects of RV apical pacing have been widely acknowledged [114,115]. Positioning the RV lead in a septal position has been associated with beneficial hemodynamic effects [116]. Early work evaluating non-apical RV pacing conducted by Khan et al. [117] showed that LV remodeling rates were unaffected by RV lead position, while Kutyifa et al. [118] also highlighted a higher risk of ventricular arrhythmias. The SEPTAL CRT study randomly assigned patients to receive either a septal or apical RV lead and demonstrated no significant difference in clinical outcome [119].

It is worth noting that there is little consensus on a universal optimal site of LV lead deployment and instead, an individualized approach which takes into account etiology, tissue characterization, and the underlying electrical substrate appears to yield the greatest benefit [120]. Similarly, patient-specific RV lead placement may represent a superior strategy, particularly when faced with limited viable LV pacing sites due to the anatomical constraints associated with transvenous, epicardial CRT. In a pilot study of seven patients, Kumar et al. observed that patient-specific RV lead placement guided by real-time assessment of the cardiac output resulted in significant acute hemodynamic improvements [121].

Electrical latency appears a useful marker when looking to identify the optimal LV pacing site [19,20], and a similar approach can also be used when looking to optimize RV lead position. In the INTER-V study, the measurement of paced RV-LV interlead electrical delay predicted mid-term CRT response [122]. In a blinded, randomized controlled trial which prospectively allocated patients to receive CRT with either RV apical pacing or RV pacing at a site guided by maximal electrical separation (MES), individualized RV lead placement was associated with increased rates of echocardiographic response [123].

## 6. Site selection during LV-only pacing

LV-only pacing has also been proposed to avoid the negative sequelae associated with RV pacing by preserving intrinsic conduction via the right bundle branch. Different approaches to LV-only pacing have been assessed for both epicardial and endocardial CRT.

### 6.1. LV-only epicardial pacing

Several comparative studies assessing LV-only epicardial (LV_EPI_) pacing timed to coincide with intrinsic RV activation have been performed. LV_EPI_ pacing has been shown to be associated with non-inferior outcomes [124–126], with some studies showing trends towards superior LV remodeling [127] and improvements in left ventricular ejection fraction [128]. In all these studies, the LV lead was empirically placed in a lateral or  posterolateral target vein and as suchit is impossible to predict the implications of delivering LV_EPI_ pacing at an alternative site.

### 6.2. LV-only endocardial pacing

LV-only endocardial (LV_ENDO_) pacing has also been investigated. The activation of the LV endocardium during CRT is associated with a reduction in both LV and BiV activation time [129]. This is in part explained by the shorter activation path length but also earlier activation of fast-conducting endocardial tissue, which possesses a higher conduction velocity. LV_ENDO_ pacing was associated with greater improvements in AHR than could be achieved using conventional, transvenous CRT (BiV_EPI_) [130]. Only BiV endocardial CRT (BiV_ENDO_) proved capable of yielding a similar hemodynamic improvement.

Much attention has been traditionally focused on correcting the underlying electromechanical delay associated with LBBB by delivering LV stimulation at the site of latest activation, typically identified in the posterolateral wall. An alternative approach is to try and replicate the activation sequence observed during normal sinus rhythm, where activation occurs initially at the left mid-septal endocardium [131,132]. When assessed, activation at this site results in an almost identical temporospatial activation envelope to that observed during normal sinus rhythm, especially when compared to BiV_EPI_, LV_EPI_, and RV_ENDO_ pacing [133]. This can be achieved via a transvenous approach, using a bespoke delivery mechanism incorporating a custom pacing lead which is introduced transvenously into the RV and positioned against the RV septum, before being deployed through the interventricular septum until the left ventricular septum is reached but without perforating the LV septum [134]. One major benefit of this approach is it negates the need for long-term anticoagulation, typically associated with lead-based LV_ENDO_ pacing.

LV_ENDO_ septal pacing can achieve a hemodynamic performance similar to that observed during normal sinus rhythm in patients with preserved LV function [134]. The effects of LV_ENDO_ septal pacing have also been assessed in a small series of patients who fulfill the current criteria for CRT implantation. A combination of RV_ENDO_ and LV_ENDO_ septal pacing achieved the greatest improvement in cardiac stroke work, suggesting that while the septum may represent a potential location to deliver stimulation in patients with impaired LV function, LV _ENDO_ septal pacing alone may not be sufficient to achieve optimal resynchronization [135].

## 7. Multi-modality imaging and image fusion technology

### 7.1. Multi-modality imaging

A novel approach to site selection incorporates the fusion of two differing imaging modalities via multi-modality imaging in order to maximize the reliability of the acquisition. Bertini et al. describe an excellent approach aimed at targeting late mechanically activating, viable tissue [136]. Patients first underwent a CMR scan where areas of the myocardial wall displaying of >75% LGE were excluded. The next stage of the ‘CRT Team’ approach involved the use of 2D speckle-tracking echocardiography which assessed global LV longitudinal strain in order to highlight the most delayed area between non-fibrotic segments. The highest frequency of reverse remodelling at six months (93.1%) was observed in the 58% of patients where the final lead position was concordant with the prespecified optimal site [136].

Another novel approach employs the fusion of pre-procedural CMR imaging with computer modeling to predict the optimal pacing site. In this series, a 3D navigation model was designed to rank the available sites for LV and RV lead deployment. Sites were graded to ensure the LV lead was directed into the segment with the lowest scar burden which exhibited the greatest mechanical delay while also maximizing the geographic distance between the LV and RV pacing sites. The RC lead tip was directed to the area with the lowest scar burden. The optimal location for the RV lead tip was assigned first followed by the preferential LV pacing site. At follow-up, 74% of patients met the predefined echocardiographic criteria of a responder despite the fact that lead implantation was informed purely by fluoroscopy and visual assessment of the 3D models, and real-time guidance was used [137].

### 7.2. Image fusion and guidance technology

Optimal site selection can only be achieved when used in conjunction with a targeting system which can identify and inform pacing electrode deployment in real time during implantation. The use of fluoroscopy alone to facilitate targeted electrode deployment is challenging given the radiolucency of the cardiac silhouette and high variability in the rotation of the left and right sided chambers relative to one another. When previously evaluated, concordance between final fluoroscopic LV lead position and CT images was only observed in 35% of patients [138]. In over half of the cases studied, LV lead deployment had actually occurred in an adjacent segment, although this is hardly surprising given the relatively small size of an individual myocardial segment (order of magnitude, cms). As such, site selection and X-Ray co-registration are essential in order to ensure optimal electrode deployment.

### 7.3. Image fusion with fluoroscopic CS balloon venography

Both the TARGET and STARTER studies showed the benefits of targeting lead deployment at the site of LMA, defined using TTE [13,16]. Unfortunately, in the STARTER study, it was only possible to deploy the lead at the target segment in 30% of patients due to issues with coronary venous anatomy and lead stability. Even in recent work where CMR was used to define the optimal pacing site, concordant LV lead positioning was only achieved in 52% of cases [93]. One approach to facilitate site selection at an achievable location subtended by a tributary of the CS is to evaluate both mechanical activation and CS anatomy. This can be achieved by fusing TTE-derived 3D echo data with fluoroscopic CS balloon venography [14]. Use of this image guidance tool resulted in an LV reverse remodeling rate of 81% of patients, where concordance between final LV lead position and the site of LMA was confirmed. While the use of coronary venous anatomy helps pre-procedural planning, this data was acquired via an additional invasive catheter study.

**Figure 3.**
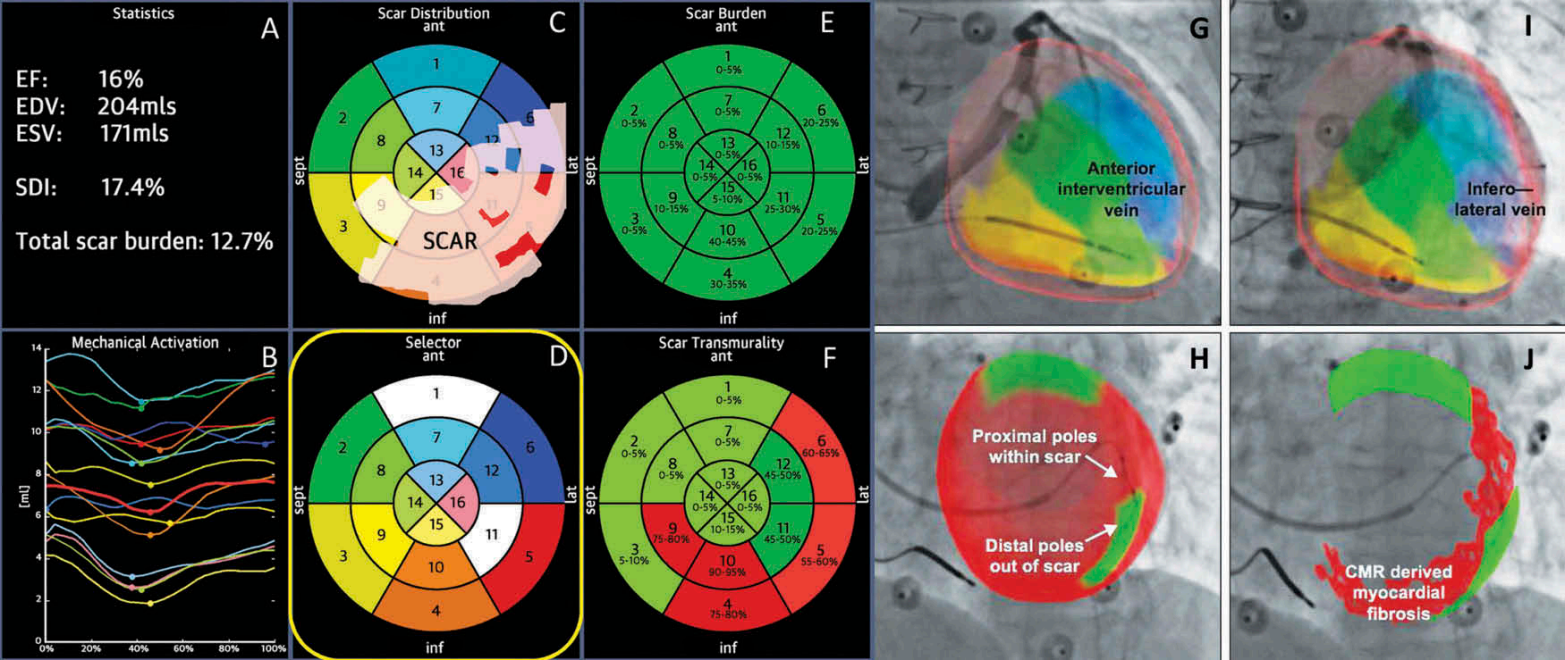
(A**)** (Top left) Anteroposterior venogram with overlay of CMR-derived epicardial/endocardial shell with 16-segment American Heart Association model showing an anterior interventricular vein. The 3D CMR-derived shell has the same colors as displayed in the guidance platform as shown in Figures 2 and 3. Infero-septal, antero-septal, and anterior segments are colored in yellow, green, and blue, respectively. (Top right) left anterior oblique (LAO) 20 venogram with automated rotation and alignment of the 16-segment model with the x-ray. Inferolateral veins are demonstrated. (Bottom left) LAO 40 projection. The positioning of a quadripolar left ventricular lead into a preselected target segment (green). (Bottom right) LAO 40 projection, alternate view with CMR-derived scar distribution (red). Attempted positioning and pacing using left ventricular poles out of regions of scar. Reproduced with permission from Elsevier due to the creative commons license. (B) This display screen is seen following the processing of the CMR data set and is mimicked on the large screen in the catheter laboratory. Total scar burden calculated as a mean of all myocardial segments. (Top middle) Scar distribution denoted in grey upon an American Heart Association 16-segment model. (Top right) Scar burden (% scar per myocardial segment volume), displayed in 5% ranges. (Bottom right) Scar transmurality demonstrating the mean transmurality from endocardium to epicardium. Those segments >50% transmural myocardial fibrosis are also denoted in red. (Bottom left) Mechanical activation curves for the 16 segments, corresponding to the colors shown in the middle panels. Endocardial tracking of the left ventricle provides absolute changes in the volume per segment (ml, y axis) over the cardiac cycle (0% end diastole, 30% to 50% end systole, 100% end diastole). Because these are absolute volume changes, the apical segments are always at the bottom because they have a smaller start and end volume. When the user hovers over a segment in the top middle panel, the associated volume time curve appears in bold; in this case, the target posterolateral segment is shown. (Bottom middle) Target selection panel. Upon reviewing the scar location, burden, transmurality, and mechanical activation curves, target segments are chosen (seen here in white; basal anterior, mid-posterolateral). EDV 1⁄4 end-diastolic volume; EF 1⁄4 ejection fraction; ESV 1⁄4 end-systolic volume; SDI 1⁄4 systolic dyssynchrony using endocardial tracking of CMR cine images in short and long axis. Reproduced with permission from Elsevier due to the creative commons license. Full color available online.

A more streamlined approach fusing peri-procedural fluoroscopic CS balloon venography with CMR imaging has also been developed [139], see Figure 3(a). A major benefit of this system is the integrated nature of the GuideCRT platform (Siemens Healthineers, Erlangen, Germany). Image processing is automatic with manual verification. A quantitative analysis of LGE is exported including information on scar location, burden, and transmurality. In addition, regional motion analysis of volume vs. time is plotted via endocardial tracking for each of the 16 myocardial segments, allowing identification of the latest activating region, see Figure 3(b). Image processing has now been accelerated to the stage (25 ± 8 min) that the patient can undergo a CMR immediately prior to their CRT implant, and by the time CS venography has been performed, image co-registration can be performed without delay.

**Figure 4.**
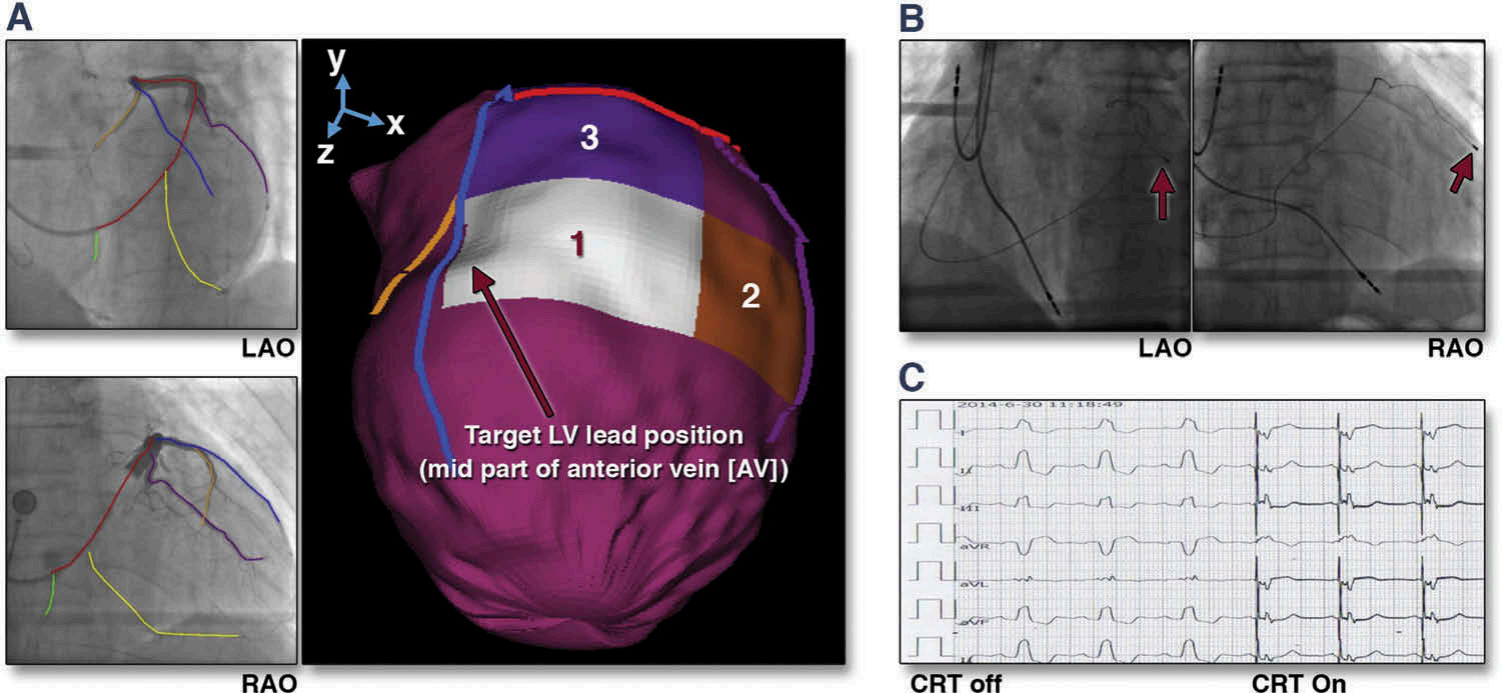
(A) Target venous site for LV lead placement. Major LV veins were drawn on fluoroscopic venograms, reconstructed to a 3D structure, and fused with SPECT LV epicardial surface. The mid part of AV (blue line) was aligned with the optimal segment (white segment), and so was targeted for lead placement. (B) Post-implant fluoroscopy. The LV lead was placed using the guidance in (A). The post-implant images show that the LV lead (red arrows) was on target. (C) Post-implant electrocardiogram. The QRS duration decreased from 168 to 140 ms immediately after the cardiac resynchronization therapy (CRT) device was turned on. RAO 1⁄4 right anterior oblique. Reproduced with permission from Elsevier.

Nuclear perfusion imaging can also usefully delineate areas of viable (non-scared) myocardium which display late activation [140]. Again as coronary venous anatomy is not delineated on SPECT imaging, geometric alignment, landmark-based registration, and vessel-surface overlay were used to fuse the 3D venous anatomy with the epicardial mesh derived from the SPECT images [141], see Figure 4.

### 7.4. Image fusion and guidance technology incorporating CT-derived CS venography

**Figure 5.**
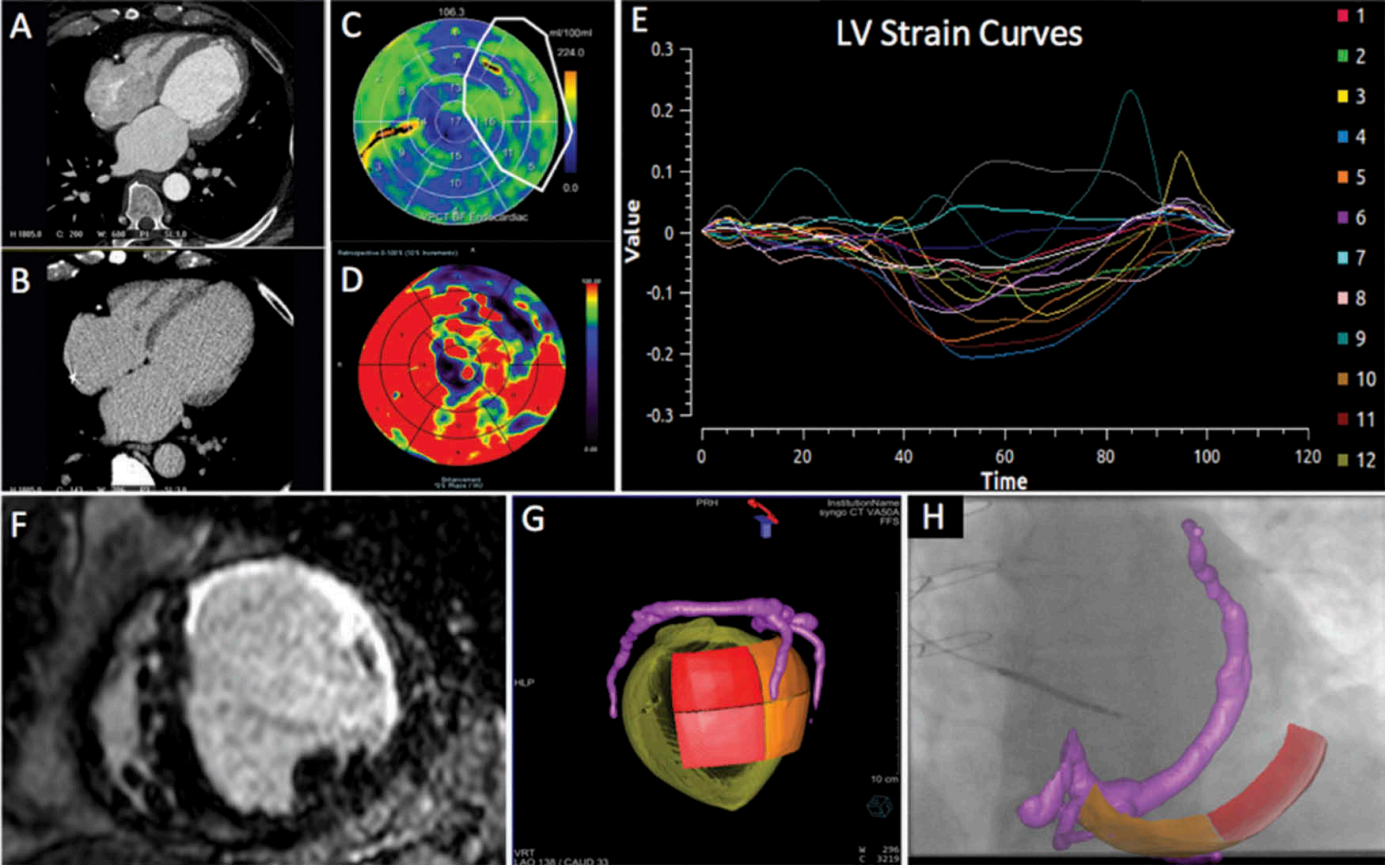
Series of (Dual Energy CT) DECCT-derived scar and image overlay of the coronary sinus and optimal target segment derived from CT strain measurements from one patient: Retrospective CCT demonstrating calcification in a left anterior descending (LAD) and circumflex territory infarct (A). Dual-energy CCT demonstrating subtle ventricular scar in the LAD and circumflex territory (B). Late iodinated enhancement plotted on American Heart Association (AHA) 17-segment bull’s-eye plot suggesting scar in the LAD and circumflex territory but also artifact from an existing RV pacing lead in the basal to mid-antero-septum (C). First pass iodine uptake plotted on an AHA 17-segment bull’s-eye plot showing what we believe to be residual iodine predominately in the LAD and circumflex territory (D). CCT-derived dyssynchrony curves calculated by myocardial strain (E). Cardiac magnetic resonance short axis image of the mid-LV showing late gadolinium enhancement of the same patient taken 2 years prior to any device implantation for comparison purposes (F). Pre-procedure DECCT-derived coronary sinus segmentation fused with latest mechanical activating segments determined from DECCT-derived strain (G) co-registered and overlaid onto live fluoroscopy using fusion software (H). Reproduced with permission from Oxford University Press.

While it is possible to visualize the CS using CMR [142], direct imaging of the sub-branches can be difficult to consistently achieve. CT, however, is capable of accurately delineating the coronary venous tree with submillimeter spatial resolution via rapid acquisition, 3D, isotropic, whole heart data sets [143]. Co-registration between pre-procedural CT-derived volumetric data sets and intra-operative 2D image acquisitions is more straightforward and can be performed using both a feature-based and an intensity-based method [144]. Work evaluating the use of DSCT to both informs site selection and identifies an overlying tributary of the contrary sinus subtending this area is currently ongoing [145], see Figure 5.

## 8. Expert commentary

Transvenous, epicardial CRT remains the optimal therapy for those patients who exhibit a remediable underlying substrate and whose anatomy is amenable to allow adequate correction. The constraints of the CS anatomy have forced the exploration of novel means or performing resynchronization pacing and of these endocardial CRT holds the most promise. Endocardial LV pacing results in a more physiological, endocardial to epicardial activation pattern, a greater reduction in total LV activation time (LV_TAT_) and an improved hemodynamic performance (greater increases in LV dP/dt_max_) in both the LV [129,146] and RV [147]. It is also associated with a higher implant success rates, less phrenic nerve stimulation, and far greater access to different pacing sites. The optimal LV endocardial pacing site displays marked inter- and intra-patient variability and while some argue that any endocardial pacing site will invariably prove superior to epicardial stimulation, research has shown that suboptimal BiV_ENDO_ CRT can achieve hemodynamic improvements inferior to those associated with empirically positioned BiV_EPI_ CRT [100]. As such, while endocardial pacing affords the freedom to perform LV stimulation at a customizable location, site selection is even more critical in order to maximize the potential benefits associated with this technique.

Tissue characterization remains an integral parameter when looking to optimize CRT delivery. Targeting viable tissue is clearly beneficial and this can be achieved using a variety of modalities. Undertaking a direct head-to-head comparison of these diagnostic approaches would aid the development of future LV lead guidance systems. While it is clear targeting viable myocardium is beneficial, it remains to be seen precisely how the presence of discrete areas of scar affects the location of the optimal pacing site.

Assessing mechanical dyssynchrony can also be achieved through a variety of modalities;however, the prospective evidence proving dyssynchrony assessments can improve patient selection for CRT is lacking [86,87]. The use of CMR-based dyssynchrony indexes may offer a more reliable solution [18]; however, larger-scale, prospective, randomized validation of these techniques is required before they can be comprehensively endorsed. In the meantime, mechanical dyssynchrony assessments aimed at identifying viable sites exhibiting late mechanical activation do appear to aid LV site selection. It is not clear whether this approach is superior to targeting sites of electrical latency or even whether these two sites are synonymous [112]. Both Q-LV [19] and LVLED [20] appear particularly useful markers to aid site selection. Neither technique requires sophisticated electroanatomical mapping or additional pre-procedural image processing and instead can be calculated by the operator at the time of LV lead deployment. A more integrated approach is likely to be necessary, however, than simply targeting the site of LEA, particularly in ischemic patients. Instead, the focus should be on targeting a site which displays late activation in the absence of myocardial scar. Narrowing of the paced QRS during pacing appears a less reliable marker of the optimal pacing site, and we would urge caution with this approach given the potential for electromechanical uncoupling. Optimizing the RV pacing site by identifying electrical delay also appears to be beneficial [123], especially when revising a previously implanted system where the position of the LV lead is relatively fixed.

Another novel approach to CRT involves attempting to selectively capture the His-Purkinji network of fast-conducting fibers via LV-only pacing. Rather than trying correct underlying electromechanical dyssynchrony, this approach aims to replicate healthy intrinsic BiV depolarization. New delivery mechanisms allow stimulation of the LV septum via transvenous access [134] meaning this approach may well become increasingly widespread. Whether similar results can be achieved through selective His capture in the RV remains to be seen, although initial studies of this promising pacing modality appear promising [148].

Site selection remains only as good as the adjunctive guidance system given the technical limitations of spatial orientation using conventional fluoroscopy [138]. Co-registration between 2D and 3D modalities is challenging with the simplest solution integrating pre-implant imaging data with peri-procedural CS balloon venography. However, these systems are unable to determine whether a suitable tributary of the CS subtends the target segment until the procedure has begun highlighting a limitation of the transvenous, epicardial CRT. Guidance systems, which can accurately visualize the entire CS network pre-procedurally, allow decisions to be made regarding not just the most appropriate target but also the most suitable means of targeting this area.

## 9. Five year view

Transvenous epicardial CRT will continue to function as the default strategy for the delivery of CRT. Pre-procedural imaging and modeling [149] will,however, allow physicians to identify those patients who may benefit from an alternative pacing strategy, either due to a lack of suitable targets or recognition that the target area cannot be accessed via the epicardial coronary venous system. More widespread recognition of cases where this is the case will result in greater use of endocardial pacing, and in particular leadless endocardial pacing systems such as the WiSE-CRT system (WiSE-CRT System, EBR Systems, Sunnyvale, California) which have proved safe and effective and do not require long-term anticoagulation [150]. Growth in endocardial pacing will mandate the increasing use of site selection and guidance given the optimal LV endocardial pacing displays such a high degree of variability.

The WiSE-CRT system has been developed with transfemoral arterial access in mind; however, arterial access complications remain an issue and given the EP community’s familiarity with transseptal access, transvenous access [151] will become increasingly derigor. In addition, entire pacing systems will likely become leadless with atrial and right ventricular activation coordinated via communication between the various components.

CMR remains the pre-eminent imaging modality for assessing both tissue characterization and mechanical activation, and the increasing experience with ultrahigh field CMR will inevitably result in improved image quality [152,153]]. Greater spatial resolution will allow detailed visualization of the entire coronary venous tree allowing pre-procedural planning to be performed entirely from one, non-ionizing imaging modality.

Other non-invasive imaging techniques which may become increasingly adopted are ECGI and Holographic imaging systems. ECGI allows an assessment of tissue characterization and can be used to locate areas exhibiting late electrical activation. Refinement of body surface mapping systems now means that instead of multiple electrode vests, a more straightforward ECG belt [154] can derive information on local electrical delay in order to guide LV lead implantation. Holographic imaging systems like the Holoscope (Real View Imaging, Yokneam, Israel) allow users to interact directly with a live 3D digital hologram. Physicians can manipulate the image (rotating, slicing, measuring, and marking) [155] fostering a greater understanding of the patients own unique anatomy.

## Key issues

- Transvenous, epicardial CRT remains an excellent therapy for those patients with right substrate and the right anatomy allowing this substrate to be corrected.
- The constraints of CS anatomy have forced the exploration of novel means of performing CRT and of these endocardial CRT holds the most promise.
- Endocardial CRT has several advantages over epicardial CRT, but the optimal endocardial site displays marked variability, and in order to maximize benefits, site selection is critical.
- Assessment of tissue characterization is essential as viable myocardium should always be targeted. This can be achieved in a number of different ways.
- There is conflicting evidence as to whether an assessment of mechanical dyssynchrony can aid in patient selection for CRT over and above current guideline indications, however, targeting sites exhibiting late mechanical activation appears useful.
- Targeting late electrical activation also appears a promising strategy. Q-LV and LVLED appear very useful when looking to identify the optimal site. The site of latest activation is not consistently associated with the optimal hemodynamic improvement. Instead, a position which displays late but not excessively late activation appears the most beneficial.
- Narrowing of the QRS during pacing appears a less reliable marker of the optimal pacing site.
- Optimizing the RV pacing site may be of benefit, especially when revising a previously implanted system where the position of the LV lead is relatively fixed.
- LV-only pacing appears promising, particularly in those with preserved LV function. New delivery technology means both of these systems can be implanted transvenously.
- Site selection is only as good as the adjunctive guidance system. Guidance systems which integrate pre-procedural imaging with peri-procedural CS balloon venography are unable to determine whether a suitable tributary of the CS subtends the target segment until the procedure has begun. This is a limitation of the transvenous epicardial approach. Systems which can directly visualize the CS during pre-procedural planning allow decisions to be made regarding the most suitable method of targeting this area.
